# Supplementary figures and images for: Dissecting the invasion of Galleria mellonella by Yersinia enterocolitica reveals metabolic adaptations and a role of a phage lysis cassette in insect killing
Source: PLoS Pathog. 2022 Nov 18;18(11):e1010991. doi: 10.1371/journal.ppat.1010991 (PMC9718411; doi:10.1371/journal.ppat.1010991)

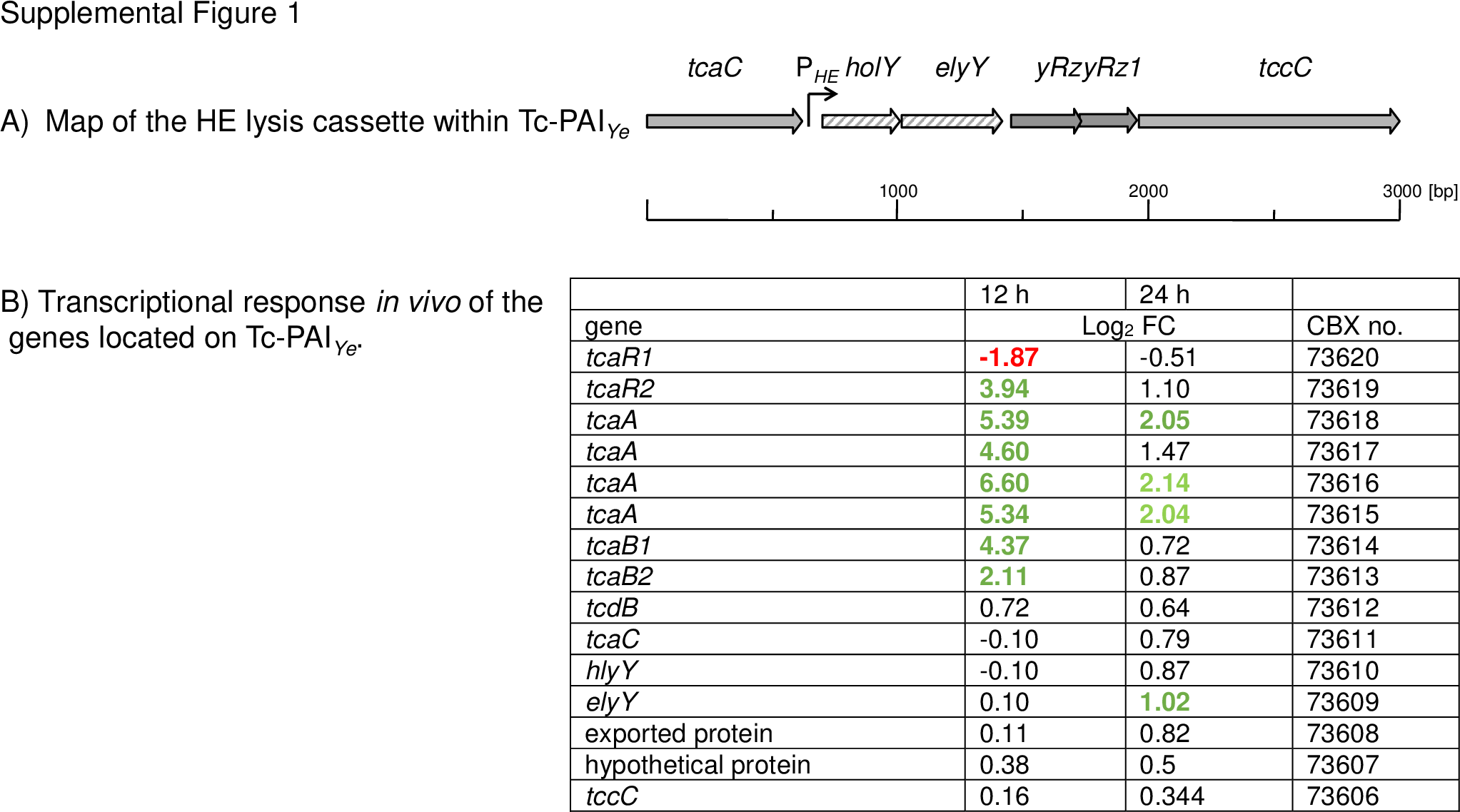

Supplement: S1 Fig — (A) map of the HE lysis cassette within Tc-PAIYe. (B) Transcriptional response in vivo of the genes located on Tc-PAIYe. (TIF) [file ppat.1010991.s001.tif]
