# Supplementary material for: Dissecting the invasion of Galleria mellonella by Yersinia enterocolitica reveals metabolic adaptations and a role of a phage lysis cassette in insect killing
Source: PLoS Pathog. 2022 Nov 18;18(11):e1010991. doi: 10.1371/journal.ppat.1010991 (PMC9718411; doi:10.1371/journal.ppat.1010991)
Supplement: S2 Table — (DOCX) [file ppat.1010991.s003.docx]

**Table S2.** Strains and plasmids used in this study.

| **Strains** | | **genotype/relevant features** | **reference or source** |
| --- | --- | --- | --- |
| *E. coli* | |  | |
|  | DH5α | F^-^ φ80Δ*lac*ZΔM15 Δ(*lac*ZYA-*arg*F) U169 *rec*A1 *end*A1 *hsd*R17(r_k_^-^, m_k_^+^) *pho*A *sup*E44 λ- *thi*-1 *gyr*A96 *rel*A1 | Invitrogen, Karlsruhe, Germany |
|  | SM10 | *lacY*, *tonA*, *recA*, Mu_c_+, *thi*, *thr*, *leu*, *supE*, RP4-2-Tc::Mu, Km^R^, λ*pir* | [1] |
| *Y. enterocolitica* | |  | |
|  | W22703 | Nal^R^, Res^-^ Mod^+^, pYV^-^ | [2] |
|  | W22703 Δ*tcaA* | W22703 mutant with a non-polar deletion of *tcaA* | [3] |
|  | W22703 ΔHE | W22703 mutant with a non-polar deletion of *holY and elyY* | This study |
|  | W22703 Δ*tccC* | W22703 mutant with a non-polar deletion of *tccC* | This study |
|  | W22703 Δ*tcaR2* | W22703 mutant with a non-polar deletion of *tcaR2* | [4] |
|  | W22703 *tcaA*::*rfp* | Gene *rfp* fused immediately behind *tcaA* via chromosomal insertion of pUTs-`*tcaA*::*rfp* | [4] |
|  | W22703 P_HE_::*rfp* | Gene *rfp* fused to the HE promoter via chromosomal insertion of pUTs-P_HE_::*rfp* | This study |
| **Plasmids** | |  | |
| pKNG101 | | Conditionally replicating vector; R6K origin, mobRK2 transfer origin, sucrose-inducible *sacB*, Str^R^ | [5] |
| pUTs-*lux*(Cm) | | Cm^R^, transposase-negative derivative of pUT mini-Tn*5 luxCDABE* Km2; suicide plasmid in *pir* negative strains | [6] |
| pKRG9 | | Derivative of suicide vector pGP704 [7]; *ori* R6K, mob^+^ (RP4), Cm^R^, Amp^S^ | Creatogen, Augsburg, Germany |
| pKD4 | | *pir* dependent, FRT sites, Kan^R^ | [8] |
| pKD119 | | Lambda red helper plasmid, Tet^R^ | [8] |
| pCP20 | | FLP recombinase plasmid, Cm^R^, Amp^R^ | [8] |
| pUTs-*rfp*(Cm) | | As above, *luxCDABE* exchanged with *rfp* | [6] |
| pUTs-`*tcaA*::*rfp* | | Last 500 bp of *tcaA* cloned in front of *rfp* within plasmid pUTs-*rfp*(Cm) | [4] |
| pUTs-P_HE_::*rfp* | | 500 bp upstream of *hlyY* cloned via *Sac*I and *Kpn*I in front of *rfp* within plasmid pUTs-*rfp*(Cm) | This study |
| pACYC184 | | p15A origin, Cam^R^, Tet^R^, | [9] |
| pACYC-*tcaA* | | pACYC184 with an *Eco*RI fragment containing *tcaA* and its promoter region, Cam^S^) | [3] |
| pACYC-HE | | Gene *holY* and *elyY* including a 500 bp upstream region cloned via *Eco*RI into pACYC184, Cam^S^ | This study |
| pBAD33 | | Expression vector with arabinose-inducible promoter, Cam^R^ | [10] |
| pBAD33-*tccC* | | Gene *tccC* cloned into pBAD33 *via* *Sac*I and *Pst*I | This study |

References:

1. Simon R, O'Connell M, Labes M, Puhler A. Plasmid vectors for the genetic analysis and manipulation of rhizobia and other gram-negative bacteria. Methods Enzymol 1986; 118:640-659.

2. Cornelis G, Colson C. Restriction of DNA in *Yersinia enterocolitica* detected by recipient ability for a derepressed R factor from *Escherichia coli*. J Gen Microbiol 1975; 87:285-291.

3. Bresolin G, Morgan JA, Ilgen D, Scherer S, Fuchs TM. Low temperature-induced insecticidal activity of *Yersinia enterocolitica*. Mol Microbiol 2006; 59:503-512.

4. Starke M, Fuchs TM. YmoA negatively controls the expression of insecticidal genes in *Yersinia enterocolitica*. Mol Microbiol 2014; 92:287-301.

5. Kaniga K, Delor I, Cornelis GR. A wide-host-range suicide vector for improving reverse genetics in gram-negative bacteria: inactivation of the *blaA* gene of *Yersinia enterocolitica*. Gene 1991; 109:137-141.

6. Starke M, Richter M, Fuchs TM. The insecticidal toxin genes of *Yersinia enterocolitica* are activated by the thermolabile LTTR-like regulator TcaR2 at low temperatures Mol Microbiol 2013; 89:596-611.

7. Miller VL, Mekalanos JJ. A novel suicide vector and its use in construction of insertion mutations: osmoregulation of outer membrane proteins and virulence determinants in *Vibrio cholerae* requires toxR. J Bacteriol 1988; 170:2575-2583.

8. Datsenko KA, Wanner BL. One-step inactivation of chromosomal genes in *Escherichia coli* K-12 using PCR products. Proc Natl Acad Sci U S A 2000; 97:6640-6645.

9. Chang AC, Cohen SN. Construction and characterization of amplifiable multicopy DNA cloning vehicles derived from the P15A cryptic miniplasmid. J Bacteriol 1978; 134:1141-1156.

10. Guzman LM, Belin D, Carson MJ, Beckwith J. Tight regulation, modulation, and high-level expression by vectors containing the arabinose PBAD promoter. J Bacteriol 1995; 177:4121-4130.
