# Supplementary material for: Dissecting the invasion of Galleria mellonella by Yersinia enterocolitica reveals metabolic adaptations and a role of a phage lysis cassette in insect killing
Source: PLoS Pathog. 2022 Nov 18;18(11):e1010991. doi: 10.1371/journal.ppat.1010991 (PMC9718411; doi:10.1371/journal.ppat.1010991)
Supplement: Attachment — Submitted filename: 128245_1_rebuttal_2432988_rgcd4q.pdf [file ppat.1010991.s005.pdf]

This is already a full revision, not a revision plan. All points were carefully addressed. TMF

July 28, 2022

RE: Review Commons Refereed Preprint #RC-2022-01555

Dear Dr. Fuchs,

Thank you for sending your manuscript entitled "Dissecting the invasion of Galleria mellonella by Yersinia enterocolitica reveals metabolic adaptations and a role of a phage lysis cassette in insect killing" to Review Commons. We have now completed the peer review of the manuscript. Please find the full set of reports below.

Reviewer #1 (Evidence, reproducibility and clarity (Required)):

In this manuscript Saenger et al. concentrate on the pathophysiological details of insect larvae infection by Yersinia enterocolitica. The authors studied the colonisation, proliferation, tissue invasion, and killing activity of the bacteria in Galleria mellonella larvae. Their study provides valuable evidence for the biological relevance of Tc toxins and a neighboring holin-endolysin cassette during establishment of Y. enterocolitica infection in Galleria mellonella larvae through the oral route. The findings of the authors provide important novel insights, that can be used for the development of Tc toxins as biopesticides.

In general, this is a nice study. The data and the methods are presented well so that they can be reproduced and the key conclusions convincing.

Unfortunately, the manuscript is sloppily written in some places, including grammatical and formatting errors. Citations regarding the structure and mechanism of action of Tc toxins are arbitrarily chosen, often taking the wrong ones and important aspects are left out. I highly recommend that the authors read the review of Roderer and Raunser 2019 that nicely describes and summarizes the molecular mechanism of Tc toxins.
Answer: We have now improved the writing of the manuscript and corrected several errors and typos. In particular, the review by Roderer and Raunser, as well as other literature in the field, is now considered and cited in the text.

The abstract ends with a speculation: "Suggesting that this dual lysis cassette is an example for a phage-related function that has been adapted for the release of a bacterial toxin" - this is likely true, but not proven in this work. What if it is used for the release of something else like extracellular DNA needed for biofilm formation (see https://doi.org/10.1038/ncomms11220)?
Answer: This sentence was carefully written as a hypothesis strengthened by the data obtained in our study. Experimental evidence for this assumption is the strong correlation of toxin and HE cassette phenotypes of mutants (see abstract), the highly conserved localisation of the cassette within Tc loci of distinct bacterial genera (see discussion for literature), and the synchronic regulation of both the toxin and the lysis genes (manuscript in preparation). Moreover, strain W22703 is unable to form biofilms in contact with invertebrates (Spanier et al., AEM 2010). There, also in accordance with other reviewers, we would like to keep this statement in the text. However, to address this interesting point, we now mention the finding of Turnbull et al. in the discussion (see last paragraph).

In addition to that, several outstanding issues must be addressed:

1. Line 45 3-D structural analysis of the tripartite Tc suggests a 4:1:1 stoichiometry of the A, B and C subunits, with the A subunit forming a cage-like pentamer that associates with a tightly bound 1:1

sub-complex of B and C. This is wrong. The stoichiometry is 5:1:1 and the structure is not a cage. The statement was taken from citation 3. However, citation 3 should not be used, since the stoichiometry as well as the structure that was determined there is wrong. Use Landsberg et al. 2012 PNAS, Gatsogiannis et al. 2013 Nature instead.
Answer: We apologize for misunderstanding the literature. Reference Lee et al. was removed here, and the two papers plus Meusch et al. (Nature, 2014) are now cited. The stoichiometry was corrected, "cage" was removed.

2. "Few bacteria are known to successfully colonize and infect invertebrates" - needs a reference.
Answer: This was modified to "Several bacteria…", and we cite the recent paper by Weber and Fuchs (in press) that in Table 7g lists more than 40 bacterial species pathogenic towards insects.

3. "Their oral insecticidal activity is comparable to that of the Bacillus thuringiensis- (Bt)- toxin" - reference missing.
Answer: The reference is now cited (Bowen et al., Science 1998). Please see the last paragraph of the paper.

4. "Type a, type b and type c" subunits is not usual for the literature. Please use TcA, TcB, TcC. A-, B-, and C-components should be abbreviated as TcA, TcB and TcC respectively in order to be in line with recent literature on the topic.
Answer: This was corrected accordingly.

5. Is TccC an ADP-ribosyltransferase or does it have a different biochemical activity?
Answer: This is unknown with respect to the Tc of *Y. enterocolitica*. In the introduction, we now refer on *P. luminescens* and do not further attribute such a function to the TcC of *Y. enterocolitica*. In the abstract, we replaced "ADP-ribosylating" with "toxic".

6. "The toxic and highly variable carboxyl-terminus of TccC that has recently been demonstrated to ADP-ribosylate actin and Rho-GTPases" - this is only certain for TccC3 and TccC5 from P. luminescens. There are many such C-termini, called HVRs which have not had their activities determined yet, see here: https://doi.org/10.1371/journal.ppat.1009102
Answer: We agree and cite this article. See also the response to comment 5 above.

7. "is probably followed by receptor-mediated endocytosis" - more recent references exist for the receptor binding of Tc toxins.
Answer: We added two references pointing to glycans as receptors of the Tc (line 52).

8. "A pH decrease then triggers the injection of a translocation channel formed by the pentameric TcaA subunits into the endosomal vacuole, followed by the subsequent release of the BC subcomplex into the cytosol of the target cell" - this again is incorrect. Please read the above mentioned review and correct this passage accordingly.
Answer: We agree. This phrase was rewritten to "The attachment of the Tc to the host cell membrane is either followed by receptor-mediated endocytosis or release of the ADP-ribosyltransferase into the target cell {Landsberg, 2011 #738;Sheets, 2011 #742}{Meusch, 2014 #788}. In a pH-dependent manner, the TcA translocation channel injected into the membrane of the host cell. Conformational changes then allow the toxic component to be released into the translocation channel of TcA and from there into the cytosol {Meusch, 2014 #788}{Roderer, 2019 #871}." (Lines 51-56)

9. What is meant by "environmental Yersinia species"?
Answer: This was corrected to "…and in *Y. mollaretii*."

10. In the relevant W22703 pathogenicity island sequence

(https://www.ncbi.nlm.nih.gov/nuccore/AJ920332) previously submitted by the same group, something odd is going on with the TcA component: it appears to be split into three polypeptides (tcaA, tcaB1, tcaB2). In the manuscript you state TcA is made up from only tcaA and tcaB. Could you please address this?
Answer: Shotgun sequencing was performed 15 years ago, and mapping revealed a frameshift within tcaB that resulted in the split annotation of tcaB. Even if this frameshift is not the result of a sequencing error, it obviously does not result in Tc inactivation. As this frameshift was not identified in most other Tc-PAI of yersiniae, we assume our statement to be correct.

11. "And their products were recently shown to act as a holin and an endolysin, respectively" - missing reference.
Answer: The reference is now cited (Springer et al., JB 2018).

12. "Its Tc proteins are produced at environmental temperatures, but silenced at 37{degree sign}C." versus "Remarkably, HolY and ElyY lyse Y. enterocolitica at body temperature, but not at 15{degree sign}C". Please address the issue that HolY/ElyY lyse the bacteria at temperatures where Tc proteins are not produced.
Answer: In the absence of *in vitro* conditions activating the HE gene cassette, we used the pBAD system to artificially overexpress the two genes and showed cell lysis at 37°C, but not at 15°C (Springer et al., JB, 2018). This finding points to a lack of cell lysis as prerequisite for TC release and strengthens the hypothesis of a new secretion system as now corroborated in the last paragraph of the discussion. To avoid confusion of readers, the sentence was removed from the manuscript.

13. "Nematodes, which are easily maintained in the laboratory without raising ethical issues, have successfully been used to identify virulence-related genes in a broad set of bacterial pathogens" - what is the relevance of this for the current manuscript?
Answer: Invertebrates are introduced here as infection models. Nematodes are mentioned here for two reasons: yersiniae are nematocidal due to the Tc, and their immune system is less elaborated than that of *G. mellonella*, thus explaining its preferred use as insect model. We shortened the sentence by deleting the phrase in commas.

14. Fig. 1C - no description is given for the labels 1-8.
Answer: This is given below figures 1E-H. The labels are valid for all figure panels to ease reading.

15. "The hemolymph of these cadavers was found full of Y. enterocolitica cells" - injected CFUs are provided here, but not final CFUs in the cadavers (although referred to in a later section). Please address this.
Answer: These were preliminary experiments to identify the optimal infection dose. Hemolymph content was plated, but cell numbers in the hemolymph were not enumerated. This sentence therefore now reads: "…and the hemolymph of these cadavers contained Y. enterocolitica cells." (lines 113-114).

16. What is the inducing agent used for pACYC-tcaA and pACYC-HE? Why would "slight leakiness of the pBAD-promoter" make pBAD-tccC non-inducible? Were colonies taken from the cadavers to verify that the bacteria still contained these plasmids?
Answer: Within pACYC, the genes *tcaA* and *hlyY*/*elyY* (HE) are under control of their own promoters as indicated in Table S2. In general, pACYC vectors are often and successfully used for complementation due to middle copy number.
This now reads "Due to the slight leakiness of the pBAD-promoter, arabinose was not added to further induce *tccC* transcription." (lines 133-134).
The presence of the plasmids *in vivo* was confirmed by periodic plating on selective and non-selective plates, not revealing differences in cell numbers.

17. Can the authors please address the TD50 of 1.83 days for W22703 ΔHE/pACYC-HE versus 3.67 days for WT bacteria? This would mean that the former kill larvae twice as fast as usual. I would not call this "did not significantly differ in their insecticidal activity".
Answer: This statement is indeed not very intuitive given the variations of the TD50-values. However, the significance here (and elsewhere in the text) is based on a statistical calculation. For the Kaplan-Meier-plot, we used an application (K.T.Bogen, Advances in Molecular Toxicology, 2016; Exponent Health Sciences, Oakland, CA, United States; Johann Kummermehr, Klaus-Rüdiger Trott, Stem Cells, 1997; Academic Press, London, San Diego) based on all data of a graph. However, to consider this point and to not confuse the readers, the phrase was modified to "…did not significantly differ in their insecticidal activity from that of the parental strain W22703 after one week, demonstrating…" (lines 135-138).

18. Fig. 2 is missing survival data for larvae infected with tcaA, HE, and tccC KO bacteria.
Answer: These data are shown and are equal to the LB-control, e. g. the survival rate of larvae infected with strains W22703 lacking HE, *tcaA*, or *tccC* were 100%.

19. "And a slight colouring of some of the larvae from one h p.i. on (data not shown)" - best show the data or remove this statement.
Answer: Although we observed this phenomenon regularly, monitoring and documentation cannot be provided and would not substantially strengthen the manuscript. We therefore deleted this phrase.

20. The infection of larvae by W22703 ΔtccC/pBAD-tccC is missing, the other bacterial variants are present. Please address this.
Answer: Infections with W22703 Δ*tccC* are not shown to not overload the figure, please see the panel below. W22703 Δ*tccC*/pBAD-*tccC* infections have not been documented by photos. Figure legend 3 now reads "Infections with W22703 Δ*tccC* and Δ*tccC*/pBAD-*tccC* are not shown."

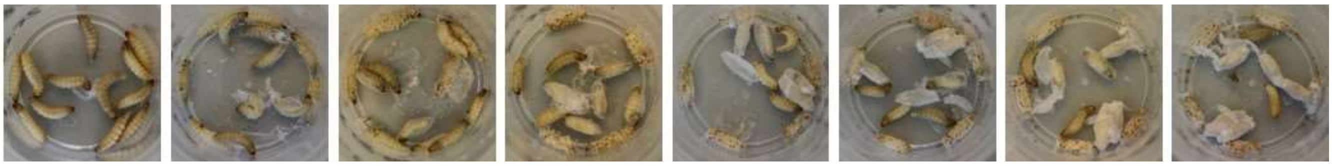

21. "initially proliferated from an application dose of 4.0 × 105 CFU and 4.0 × 105 CFU, respectively, to 2.2 × 106 CFU and 2.8 × 106 CFU, but could not be detected from day three on. This finding strongly suggests that TcaA is involved in adherence to epithelial cells and thus in midgut colonization". Please address the "initially proliferated" (which day post-infection?), their elimination from the larvae (how, why?), why the tccC KO bacteria were more virulent than tcaA KO bacteria, and where the suggestion about TcaA involvement specifically in adherence comes from.
Answer: "initially proliferated" was rewritten to "proliferated within the first day p.i.". (line 163)
Elimination: This now reads "…was completely absent six days p.i., probably due to passage through the gut followed by excretion". (lines 161-162)
In our view, the *tccC* knockout mutant is not more virulent than W22703 Δ*tcaA* (se Fig. 2), but replicates during the first day post infection, whereas the cell numbers of the tcaA KO mutant strongly decrease already within the first 24 h p.i.. This prompted us to speculate that Tc is involved in two infection steps, e.g. adherence and hemocyte inactivation. For clarity, this sentence was modified to: "This discrepancy suggests that TcaA is involved in adherence to epithelial cells and thus in midgut colonization, without requiring TccC." (lines 165-166)

22. In Fig. 4, the CFUs for W22703 ΔtccC/pBAD-tccC are essentially the same as for the other rescued

KOs and WT, while in the text a point about weaker growth is made. Is this justified? Also, even though the CFU data is present here, data on infection of larvae by W22703 ΔtccC/pBAD-tccC is missing unlike the other bacterial variants. Please explain.

Answer: We agree that this part of the results is misleading. We want to stress that the complementation very well restores the phenotype of the wildtype. The weaker growth of Δ*tccC* may be due to the distinct vector system used here. This part was there shortened and rephrased to: "When larvae were infected with 4.0×10^5 CFU of the Δ*tcaA* and ΔHE mutants, and with 1.4×10^6 CFU of strain W22703 Δ*tccC*/pBAD-*tccC*, all of which carrying the deleted genes on recombinant plasmids, the bacterial burden at days one to six p.i. increased approximately to that of the parental strain W22703 applied with 9.0×10^5 CFU, indicating a successful complementation of the gene deletions."
" (lines 166-170).
Missing data on W22703 Δ*tccC*/pBAD-*tccC* infection in Fig. 3, please the answer to point 20 above.

23. Fig. 6b - The presence of an anti-RFP signal is not obvious in any of the bottom row images. The top row images are missing the same kind of annotation provided for Fig. 6a, without which non-histologists will find understanding the figure difficult.
Answer: The anti-RFP signal is visible only on the left photo of the bottom panel, and not in the other three photos as explained in the text. We understand that the signals are not very strong, but they are visible on the screen.

24. "In the absence of the lysis cassette, however, TcaA::Rfp was not detected despite the presence of W22703 ΔHE tcaA::rfp cells." + "To test whether or not the promoter of the lysis cassette is active in vivo, we infected G. mellonella larvae with strain W22703 PHE::rfp. Although Y. enterocolitica cells densely proliferated within the hemolymph (FIG. 6B), no staining signal that would point to the presence of TcaA was obtained, possibly due to no or weak PHE activity." Does this mean that without HE, tcaA does not express?
Answer: No, we performed Western Blots showing that TcaA is detected in cells lacking HE. Therefore, a negative feedback regulation (e. g. increasing intracellular amounts of TcaA repress its own transcription) can be excluded. This is also in line with the low transcriptional activity of the lysis cassette in vivo (new Fig. S1B).

25. "These data suggest that the HE cassette is responsible for the extracellular activity of the insecticidal Tc." Please explain how the preceding paragraph leads to this conclusion.
Answer: This was poorly written and now reads "…for the transport…" (line 224).

26. "As expected, bacterial cells, e.g. Y. enterocolitica, are visible in the hemolymph obtained from W22703-infected animals, but not in all other preparations." - which figure are the authors referring to?
Answer: We have indeed identified, but not immunostained, bacterial cells in those preparations, but they are not visible in Fig. 7. This sentence was removed. However, the presence of W22703, but not its *tc*-PAI_{Ye}-mutants, in the hemolymph is demonstrated in Fig. 6A.

27. "To delineate the transcriptional profile of Y. enterocolitica during infection of G. mellonella, we applied immunomagnetic separation to isolate Y. enterocolitica from the larvae 12 h and 24 h after infection" - do the authors store the bacteria for up to 24 h at 4 {degree sign}C, as indicated in the methods section?
Answer: Yes, the probes were stabilized with RNAlater and then stored up to 24 h to synchronize all samples of one experiment.

28. "The endolysin located within Tc-PAIYe was significantly up-regulated after 24 h, but not after 12 h, pointing to its possible role in the release of the Tc" - I could not find the endolysin in Table S1. Could the authors mark it clearly? Also, why is the holin also not upregulated?

Answer: The endolysin gene is lacking in Table S1 due to its FC=1.02. We now added a table to Fig. S1 that shows the FC values of all genes from Tc-PAI_{Ye}. The FC-value of holin gene is 0.87, thus pointing to a very slight transcription of this lysis gene as discussed, thus preventing cell death.

29. "This is in line with the fact that a T3SS is lacking in strain W22703" - Is a complete genomic sequence available for this strain, so readers could validate this statement?
Answer: The genome sequence is available, and the reference is now cited (line 358). The common virulence plasmid of yersiniae, pYV that encodes the T3SS, is missing in this strain. We do not mention here the presence of a second, but probably incomplete, chromosomally encoded T3SS in strain W22703 do not overload the manuscript.

Reviewer #2 (Evidence, reproducibility and clarity (Required)):

This is a very, very nice study as it actually describes the role of different Tc toxin components in a model infection system using an important bacterium- really for the first time in a properly controlled manner. The mutants lacking either the syringe (AB) or the bullet (C) make 'sense' for a loss of function perspective. The description of the phage cassette in loss of function is also interesting and could do with some more speculation? For example, some groups of Photorhabdus bacteria release their oral toxicity (Tc's) into their bacterial supernatants- whereas in others it remains cell associated. The likely role of this phage cassette in this process should be discussed (is cell suicide required for release?).
Answer: We now discuss the possibly role of the lysis cassette in more detail, including the possibility that a subpopulation commits cell suicide (see lines 375-396).

Reviewer #2 (Significance (Required)):

This is highly significant finding as despite all of the very elegant structural studies done on these important toxins there is still very little work in vivo. These studies clearly show the role of the different components of these ABC toxins in vivo. **It should be published with priority.**

Congratulations to the authors.

Reviewer #3 (Evidence, reproducibility and clarity (Required)):

Summary: The authors analyze the phases of infection of Galleria mellonella by Yersinia enterocolitica following forced oral feeding.
They study different phases of infection, including survival within the gut and invasion of the hemolymph. By analyzing differences in the genes up- and down regulated, they show that for example transporters for food sources from the hemocoel are regulated for making those sources available for the bacteria.

Major comments: **This is an interesting paper** demonstrating genes of Y. enterocolitica dependent for colonization, growth and crossing of the epithelial gut barrier in G. mellonella.

Major points which have to be addressed:

Introduction: line 54: the BC subcomplex is not released into the cytosol! It is only the hypervariable region (enzymatic part) which enters the cytosol. This has to be corrected.
Answer: This has been corrected accordingly.

Fig.2/3: Why have different CFU been used for the distinct bacterial strains?

This does not allow a direct comparison of their toxicity. For me the dead larvae shown in Fig. 3 are not represented in Fig 2 (data are not concordant), because of the loss before day one depicted in Fig. 2: The curves should be normalized to the same starting point (should be 100 %)?
Answer: We would like to stress here that infection doses are hard to reproduce if frozen and diluted stocks are used. We decided for overnight culture to better mimic natural conditions and controlled each culture for its viable cell numbers by plating. Moreover, we choose the infection doses in a conservative manner, e.g. the number of mutants was higher than that of the parental strain.
The data of Fig. 3 are concordant with Fig. 2 for two reasons: First, this experiments was performed in replicates with a total of 36 larvae per strain (see Fig. 2 legend), so that representative photos are shown. Second, larvae were considered dead if they failed to respond to touch, and many larvae without strong sign of melanisation were already killed.
We analysed the algorithmus of the Kaplan-Meier-plot. All graphs start at 100%, this is now mentioned in the legend. There are no data between day 0 and day 1, and a stepwise graph is essential for this plot.

Fig. 3: Why is the strain W22703 delta tccC/pBAD - tccC missing in the data set?
Infections with W22703 Δ*tccC* are not shown to not overload the figure, please see the panel below.
Answer: W22703 Δ*tccC*/pBAD-*tccC* infections have not been documented by photos. Figure legend 4 now reads "Infections with W22703 Δ*tccC* and Δ*tccC*/pBAD-*tccC* are not shown."

Minor: line 221: "the" is doubled
Answer: This has been corrected accordingly.

Reviewer #3 (Significance (Required)):

The manuscript shows the use of G. mellonella as a straight foreward method to study gene functions of pathogenic bacteria, a significant knowledge for scientists of the field.

Reviewer #4 (Evidence, reproducibility and clarity (Required)):

Summary:
Provide a short summary of the findings and key conclusions (including methodology and model system(s) where appropriate).
Answer: There are already three sections that summarize the results and the methods applied, namely the abstract, the last paragraph of the introduction, and the conclusion following the discussion. In our view, a further summary would overload the manuscript. Nevertheless, depending on the journal the manuscript will be published in, an additional authors´ summary would be provided.

Outlines proposed role of lysis cassette in oral infection of Galleria as a model insect for host pathogen interaction, data which is fortified through use of histology and RNAseq.

Introduction could extend to additional background eg Aleniz et al and other entomopathogen transcriptome data, more so other studies using Yersinia and Galleria as a model (refer references provided in the below comments)
Answer: We again carefully screened PubMed for studies in the field and added few papers. However, in vivo transcriptome analyses are still rare, as indicated by a lack of a respective investigations with the highly relevant entomopathogen *Photorhabdus luminescens*. The literature suggested by the reviewer is now cited in the introduction and the discussion (see below for details).

The strength of the paper lies in understanding the progression of the disease in the insect host as mentioned L316-317 and clearance of the bacteria via in TcaA mutant

Major comments:
- Are the key conclusions convincing?
Yes for mode of action
Fig 5 could have additional panels -this is a strength of the paper
Answer: We agree that this time course is a strength of the paper, and we carefully selected representative photos. There are several to be shown, but to our view, they are rather illustrative than providing a substantial additional value.

Fig 6 legend could better describe the observed insect components
Answer: The insect components are now indicated in Fig. 6B and in Fig. 5.

Figure 7 may be lost in PDF conversion -the figure appears un resolved? are there more high resolution photos
Answer: Fig. 7 was present in the merged PDF provided by the publisher. We used the photos with the best resolution.

- Should the authors qualify some of their claims as preliminary or speculative, or remove them altogether?
the data provided is in places rudimentary (i.e. validation of the role of the lysis cassette in virulence) and could be bolstered with the construction and use of a lysis translational reporter etc I was left unsure how the HE::rfp and TcA::rfp constructs were made. I had assumed red florescent protein however it appears an antibody is used. This needs to be clarified as I then found it hard to interpret the results.
Answer: The transcriptional PHE::rfp fusion is mentioned in the results section, but immunostaining failed probably due to a very low promoter activity (line 223). This is well in line with the transcriptome data. Please see a detailed answer how the HE::rfp and tcaA::rfp were constructed below. We applied the RFP-antibody for two reasons: first, fluorescence microscopy did not reveal clear red fluorescence in the tissue sections, and second, a TcaA antibody failed to match quality criteria for this purpose.

It appear l114-125 that their may be enough data to derive a LD50 values and or LT value at a fixed dose - if so reporting this data of interest. It may also allude as to why a 10e5 dose was selected for subsequent expts
Answer: This is an interesting point. The LD50 (dose of cells that kills 50% of all larvae) is usually not calculated in publications in this field of research, because its calculation requires a very huge separate data set that cannot be used to answer the questions addressed here. Such a dat set is not available. We published the dose-dependent toxicity of Y.enterocolitica W22703 upon subcutaneous injection, and from these data, we determined a LD50 for this strain of approximately 2 x 10^4 cells. The paper is cited in our manuscript. The 10E05 dose was selected due to our preliminary work and the reproducibility of the experimental phenotypes.

- Would additional experiments be essential to support the claims of the paper? Request additional experiments only where necessary for the paper as it is, and do not ask authors to open new lines of experimentation.
Use of lysis the reporter - discuss commonalties of the in host transcriptome with other Yersinia Galleria systems eg Paulson etc al (refer below). Are there any thoughts on the host range of this Yersinia and can this be placed in a pathogen host evolutionary context?

Answer: Paulson et al. are now cited twice in the text. The host range of *Yersinia enterocolitica* has not been investigated to our knowledge. However, its nematocidal activity has been described by Spanier et al., and *Manduca sexta* larvae, the tobacco hornworm, is also killed by W22703 (see references). Moreover, there are two copies of tccC in the genome of strain W22703 encoding the cytotoxic Tc subunit with its hypervariable C-terminus that is assumed to contribute to host specificity. This is discussed in very detail by Song et al. (see references).
Evolution: Yes, this has been addressed by Waterfield et al. 2004 (see references) where insects are hypothesized as a source of emerging pathogens. We placed our findings in the context of this article in lines 91-94 and 305-310.

- Are the suggested experiments realistic in terms of time and resources? It would help if you could add an estimated cost and time investment for substantial experiments.
Yes

- Are the data and the methods presented in such a way that they can be reproduced?
yes but I think some vector construction methodology is missing e.g. ::rfp (refer above)
Answer: The plasmids used to construct the two strains W22703 tcaA::rfp and W22703 PHE::rfp are listed in Table S2. References for details are given (Starke et. al., 2013, Starke and Fuchs, 2014). Briefly, we used a suicide vector (pUTs) carrying the gene encoding the red fluorescent protein (RFP). This vector replicates in *E. coli* helper strains such as SM10, but not in *Y. enterocolitica*. Strain SM10 is now listed in Table 2. Following conjugation, the construct is chromosomally inserted upon recombination via the fragments cloned into the plasmid. In case of *tcaA*, we cloned the 3´-end of the gene to generate a translational fusion, and in case of HE its promoter, resulting in a transcriptional fusion with the reporter RFP.

Fig 2 I am a little lost mortality seems quick on day 0 is this a result of aberrant injection damage mortality or are the authors observing a different effect across mutants through the initial 24 hours? If data available could this time plot be extended out 0-24 hours. The dash used for W222703 tcaA /TccC look similar can a different symbol be used.
Answer: The reviewer is right that the mortality is high on the first day. However, larvae monitoring for up to nine days is a standard in the literature. No data are available for a better resolution of the first 24 h that, however, were investigated in more detail in the time course of Fig. 5. Moreover, we observed changes in motility and colouring of some of the larvae from one h p.i. on (data not shown). Aberrant injection damage was avoided, and damaged larvae or larvae that not completely took up the infection solution were not further considered in the experiment. This is mentioned in lines 107-109.
A different symbol is now used for W222703 Δ*tccC* /pBAD-*tccC*.

- Are the experiments adequately replicated and statistical analysis adequate?
Yes

Minor comments:
- Specific experimental issues that are easily addressable.
- Are prior studies referenced appropriately?
Other entomopathogenic transcriptome studies could be compared to and or cross referenced (I have provided references in the response
Answer: Repetition of our answer above: We again carefully screened PubMed for studies in the field and added few papers. However, in vivo transcriptome analyses are still rare, as indicated by a lack of a respective investigations with the highly relevant entomopathogen *Photorhabdus luminescens*. The literature suggested by the reviewer is now cited in the introduction and the discussion (see below for details).

I am unsure on the use of immuno pulldown and efficiency of recovering the Yersinia using this method as opposed to direct sequencing total RNA has this method been used in other systems,
Answer: Isolating RNA from in vivo probes of infected insects encounters two challenges: first, a possible contamination with commensal bacteria, and a too high amount of host RNA that reduces the number of sequence reads. This might be the reason for the relatively low sequence depth found in related papers in the field of in vivo transcriptomics. We overcame these problems by immunomagnetic separation that is easily applicable and enriches the samples with respect to *Yersinia* cells, this is now mentioned in the results. We also cite a study (Prax et al., in which we established the protocol of IMS.

- Are the text and figures clear and accurate?
Yes though in places better naming of insect components could be listed
Answer: This was done, see above.

- Do you have suggestions that would help the authors improve the presentation of their data and conclusions?

As listed above potential use of reporters and or comparison and transcriptome analysis to other systems and an evolutionary pathogen host context (refer comments above) would strengthen the manuscript
Answer: Please see answer to comments above. We explained the use of the reporter fusions, and put the transcriptome analysis into the context of related studies.

Minor comments as per below
When first mentioned good to state the larval instar used
Answer: We used larvae of instar 5-6 according to Jorjao et al. (2018), this is now mentioned and cited in the M&M section, line 434.

l 78 lon protease? what type? this is an important SOS protease affecting many regulatory systems please clarify
Answer: This is a Lon A endopeptidase, and its function for the temperature-dependent activity of the lysis cassette has ben described (Springer et al. 2021, see references). Its relevance for the thermodependent regulation of *Yersinia* virulence has been documented by Herbst et al. (PMID: 19468295) and Jackson et al. (https://doi.org/10.1111/j.1365-2958.2004.04353.x).

l103-113 an description of the elemental tract which is depicted, perhaps this could be placed in the Fig. 1 figure legend
Answer: We agree and substantially shortened the first paragraph of the results. Relevant aspects are now mentioned in Figure legend 2, redundancies with the figure legend were removed.

l 133 use of the word larvae in place of the word animals might be more appropriate
Answer: This was corrected accordingly.

l 133 clarify delta HE mutant description when first mentioned
Answer: The abbreviation HE is now introduced in the introduction in line 74.

Lines 220-234 hard to follow mainly as I am unsure how then strains are constructed, perhaps clarify what rfp is how was it made :: demotes and insertion but yet then they seek to detect TcaA? I could not find the methodology on its or HE::rfp construction
Answer: The plasmids used to construct the two strains W22703 tcaA::rfp and W22703 PHE::rfp are listed in Table S2. References for details is given (Starke et. Al., 2013, Starke et al. 2014). Briefly, we

used a suicide vector (pUTs) carrying the gene encoding the red fluorescent protein (RFP). Following conjugation, the construct is chromosomally inserted upon recombination via the fragments cloned into the plasmid. In case of *tcaA*, we cloned the 3´-end of the gene to generate a translational fusion, and in case of HE its promoter, resulting in a transcriptional fusion with the reporter RFP.
Please see above why we used RFP-antibodies to detect TcaA.

l247 immuno-magnetic separation to isolate Yersinia - is there an efficiency behind this method, might be good to mention (I am unfamiliar with this technique)
Answer: We here repeat our answer to the point above: Isolating RNA from in vivo probes of infected insects encounters two challenges: first, a possible contamination with commensal bacteria, and a too high amount of host RNA that reduces the number of sequence reads. This might be the reason for the relatively low sequence depth found in related papers in the field of in vivo transcriptomics. We overcame these problems by immunomagnetic separation that is easily applicable and enriches the samples with respect to *Yersinia* cells, this is now mentioned in the results. We also cite a study (Prax et al., in which we established the protocol of IMS.

l313 alludes to role of Tca in hemoceol which contradicts an earlier statements in l 130 please clarify
Answer: The reviewer is right. The sentence in former line 130 (now lines 123-124) was corrected to "…suggesting that the Tc plays a main role in the initial phases of infection". This statement does not exclude its activity towards hemocytes. Moreover, subcutaneous infection is very artificial and was therefore replaced by oral application in our study to mimic natural routes of infection. This is now elaborated in more detail in the discussion (Lines 305-310).

For clarity table 1 could colour highlight (different colours) tc and lysis genes
Answer: We now added a table to Fig. S1 that shows the FC values of all genes from Tc-PAI_{Ye}.

CROSS-CONSULTATION COMMENTS
I am in agreement with all points of reviewer 1 who has a clear understanding on Tc toxin composition TcA pentamer etc. Being familiar to the field I regret I did not pick up on these errors
Answer: This has been corrected according to R1.

Point 13 agree and should possibly bring in other researchers who have used Galleria as a model. It also needs to be kept in mind that the target host for many Tcs has yet to be determined hence the importance of oral activity of this isolate
Answer: This has been corrected according to R1.

I am similarly in agreement with comments of reviewer 3

Reviewer 4 I over looked the LT50 data -- apologies but agree with reviewer 1 where WT should be the more potent strain --I still think if possible LD50 for WT would be of value more so to define its oral activity
Answer: We repeat our answer from above. This is an interesting point. The LD50 (dose of cells that kills 50% of all larvae) is usually not calculated in publications in this field of research, because its calculation requires a very huge separate data set that cannot be used to answer the questions addressed here. Such a dat set is not available. We published the dose-dependent toxicity of Y.enterocolitica W22703 upon subcutaneous injection, and from these data, we determined a LD50 for this strain of approximately 2×10^4 cells. The paper is cited in our manuscript. The 10E05 dose was selected due to our preliminary work and the reproducibility of the experimental phenotypes.

Reviewer #4 (Significance (Required)):

SECTION B - Significance

========================

- Describe the nature and significance of the advance (e.g. conceptual, technical, clinical) for the field.

Extends from work of Fuchs - research group
Extends from work of Palmer et al on lysis cassettes as potential T10SS
Extends from work off Vesga Pseudomonas and Paulson Yersinia(refs provided below) on insect transcriptomics

Of interest and possibly understated is the oral activity of enterocolitica in the insect host as mentioned L316-317 and how this might relate to the lifestyle/evolution of this microbe further elaboration here would be of interest
Answer: We agree that this is an important aspect. Therefore, we added the following sentences here: "In contrast to subcutaneous injection in the use of insect larvae as model for bacterial virulence properties towards mammals, oral application mimics natural routes of infection that in particular take place during the bioconversion of animal cadavers by bacteria, fungi, and larvae {Carter, 2007 #879}. Together with the broad cytocidal host spectrum of bacterial toxins {Mendoza-Almanza, 2020 #880}, investigation of yet neglected natural infections of invertebrates will contribute to a better understanding of microbial pathogenicity {Waterfield, 2004 #480}." (lines 305-310)

- Place the work in the context of the existing literature (provide references, where appropriate).

Relevant Transcriptome papers which could be referred to in the discussion i.e. are similar genes in play or is their a point of difference?
https://doi.org/10.1093/g3journal/jkaa024;https://doi.org/10.1038/s41396-020-0729-9;
https://doi.org/10.1099/mic.0.000311
Answer: Paulson et al. mainly address virulence factors, whereas metabolism is not uncovered. We now cite similarities with respect to hemolysis and iron scavenging. The focus of Vesga et al. is on the interaction of a plant pathogen with wheat and two insect hosts, including their transcriptome. Although metabolic details are missing, there is an interesting overlap with the paper by Vesga et al. (hemocoel as permissive environment for proliferation) and a difference (upregulation of chitinases was not observed) that are now cited in the discussion. The Alenzi paper mainly investigated the general virulence of Y. enterocolitica strain. We cite its finding on the importance of motility, thus confirming our transcriptome analysis.

- State what audience might be interested in and influenced by the reported findings.
The oral activity of enterocolitica towards Galleria of interest and an evolutionary context insect vs mammalian activity in the discussion could be provided. Potential role of TcaA in gut association
For the targeted journal I feel additional technical data is required and a broader context to other global systems (bacterial species) provided
Answer: All points were addressed carefully and in detail. We refer to our answers to points detailed above.

- Define your field of expertise with a few keywords to help the authors contextualize your point of view. Indicate if there are any parts of the paper that you do not have sufficient expertise to evaluate.
Reviewers expertise entomopathogens, their toxins and pathogen ecology
