# Supplementary material for: Dissecting the invasion of Galleria mellonella by Yersinia enterocolitica reveals metabolic adaptations and a role of a phage lysis cassette in insect killing
Source: PLoS Pathog. 2022 Nov 18;18(11):e1010991. doi: 10.1371/journal.ppat.1010991 (PMC9718411; doi:10.1371/journal.ppat.1010991)
Supplement: Attachment — Submitted filename: Second point by point answer.docx [file ppat.1010991.s006.docx]

Reviewer's Responses to Questions

Reviewer #2: The author did a great job in reviewing their manuscript. In my opinion, the manuscript is ready for publication after the following minor points have been addressed:

Final sentence of abstract: as a hypothesis, this can of course be left in. But please rephrase to show that this is based on indirect evidence. I would suggest: "..., suggesting that this dual lysis cassette may be an example for a phage-related function that has been adapted for the release of a bacterial toxin". In the current form, this statement is too strong for the data provided in the manuscript.

**Answer:** We agree and modified the sentence accordingly.

"Even if this frameshift is not the result of a sequencing error, it obviously does not result in Tc inactivation. As this frameshift was not identified in most other Tc-PAI of yersiniae, we assume our statement to be correct." - Please provide fresh sequencing data for this region of W22703. The region between TcaA and TcaB2 is only 6100 bp, which can easily be covered by a colony PCR and the resulting PCR fragment sequenced. If the TcA component core is indeed split into three subunits, this would represent a novel finding and certainly be interesting for the field.

**Answer**: The splitting of the A-subunit into the two genes *tcaA* and *tcaB* is common in all Yersinia Tc islands (Fuchs et al., BMC Microbiology 2008) and also in *P. luminescens* (Waterfield, TiM 2001). We again sequenced the region within *tcaB* to again check the frameshift resulting in *tcaB1* and *tcaB2*. We amplified a 671 bp-fragment five times independently, and sequenced each fragment. All sequences were identical and harboured the frameshift within *tcaB* as published. Given that the sequencing coverage of the shotgun genome approach was 16.5 and that we had sequenced the region 4-fold during chromosomal walking (Bresolin et al., Mol Micro 2006), we consider the frameshift to be correct, thus confirming our publications. The text now reads “…encodes two regulators and TcA (*tcaA*, *tcaB1, tcaB2*),…”.

"This is given below figures 1E-H." - I still cannot relate these to the numbers 1-8 in Figure 1A-1D. Please explain which anatomical features correspond to which numbers.

**Answer:** The numbers and anatomical features are now clearly mentioned and described at the end of Fig. 1 legend, and removed from the figure.

"The presence of the plasmids in vivo was confirmed by periodic plating on selective and non-selective plates, not revealing differences in cell numbers." - Was a non-transformed control also checked in parallel to confirm selectivity is due to the antibiotic resistance cassette in plasmids rather than the cells acquiring resistance in situ?

**Answer:** Yes, we periodically plated W22703 following *G. mellonella* infection on agar plates containing tetracycline. This was also done to control the selectivity of the antibiotics. In none of the experiments, W22703 colonies were observed, indicating that plates with active tetracycline were used. This is now mentioned in the materials and methods section (first paragraph): “Each plate pack was tested for antibiotic activity using sensitive bacterial strains.” The chance of acquiring a tetracycline resistance genes is very low due to the low number of commensal bacteria in the gut of the larvae (a sterile diet was used) and the very low probability to acquire such a determinant via horizontal gene transfer (Kurland et al., Horizontal gene transfer: a critical view. PNAS 100 (2003): 9658-9662.

"Infections with W22703 delta tccC are not shown to not overload the figure" - Please add the shown panel to the figure, this is important data.

**Answer:** This panel has been added to Fig. 3 and is now mentioned in the results.

"W22703 delta tccC/pBAD-tccC infections have not been documented by photos." - This is a shame from a consistency viewpoint in relation to the other, photographically documented data. Please change the Figure 3 legend from "are not shown" to "were not documented by photos" for the sake of clarity.

**Answer:** This was corrected accordingly.

In lines 141-152 the W22703 delta tccC/pBAD-tccC is not mentioned in comparison to other tested strains (as per line 133) - can this be fixed?

**Answer:** This strain was accordingly added to the results.

“This discrepancy suggests that TcaA is involved in adherence to epithelial cells and thus in midgut colonization, without requiring TccC.” - This would be a novel function for Tc toxins - they have so far been considered to function only as holotoxins. Perhaps this novelty should be highlighted.

**Answer**: To not overstate this finding, we stress this finding now in the discussion (lines 315-316): “The proliferation data shown in Fig. 4 as well as the transcriptome analysis allow the conclusion that TcaA enables the adherence to epithelial cells, whereas the enzymatic active subunit TccC plays a minor role here.”

In Figure 4, the error bars for W22703 delta HE, W22703 delta HE/pACYC-HE, W22703 delta tccC, and W22703 delta tccC/pBAD-tccC are missing, although the legend says the experiments were performed as triplicates. Please address this.

**Answer**: This mistake is corrected, all error bars are now shown in Figure 4.

"The FC-value of holin gene is 0.87, thus pointing to a very slight transcription of this lysis gene as discussed, thus preventing cell death". A point well worth bringing up in the discussion, as it points to "a lack of cell lysis as prerequisite for Tc release", as you say in response to point 12.

**Answer**: This is highlighted in the discussion, lines 389-391: “Moreover, the lysis cassette is very tightly repressed *in vitro…*, a finding that was confirmed by the *in vivo* transcriptome analysis in the present study that revealed slight inductions of *elyY* (log_2_ FC = 1.02) and *hlyY* (log_2_ FC = 0.87)(Fig. 1B).”

Reviewer #3: The manuscript is in general improved with greater emphasis on TcA component and per os activity. the authors have attempted to address previous responses in the responses to reviewer sections and only carried through some of these corrections into the revised manuscript.

There are still some areas that if acted on may improve the readability of the revised manuscript, in addition to this a couple of contradictory sentences (as provided below) were noted which need to be clarified. I also think a final comprehensive proofreading of the manuscript is needed to smooth out some sections.

**Answer**: We very carefully went through the whole manuscript and modified many phrases to improve grammar and writing, and to ease understanding. A marked-up manuscript file shows all changes made in comparison with the last version. The contradictory sentences have been clarified (see answers below).

Never the less the authors have clearly demonstrated the per os activity and a role of TcA in gut retention. On this note [https://www.ncbi.nlm.nih.gov/pmc/articles/PMC1230942/](https://scanmail.trustwave.com/?c=4477&d=qsDE4_IvgAOuOwceLK0FIHZZWxKuXbvI-s27W-yA-A&u=https%3a%2f%2fwww%2encbi%2enlm%2enih%2egov%2fpmc%2farticles%2fPMC1230942%2f) refer to role of a TcA in gut retention in mouse (might be good to integrate this reference).

**Answer**: This finding is now mentioned in the text (lines 77-79), and the reference is cited.

They have placed less emphasis on lysis where their transcriptome profile is only partly linked to that of the Tc cluster

**Answer**: We agree. We now better link cell lysis and Tc release in the discussion. For example, we state in lines 389-391 that the low transcription of the lysis genes correlates well with the assumption that cell lysis does not occur to measurable extent. This fits well to our conclusion that the HE cassette rather acts as a secretion system than lyses the bacterial cells. Moreover, we are going to submit a paper in which the hypothesis that cell lysis is needed for Tc release is experimentally tested (and rejected by our data).

Reviewer #3:

L78 I still think more information on Lon protease needs to be provided (this might be known to the authors but may not be known to the readers)

**Answer**: This sentence now reads (lines 90-94): “Upon deletion of the gene encoding the Lon A protease, which is involved in the thermodependent regulation of *Yersinia* virulence properties (21, 22), we observed lysis of the mutant at 15°C, indicating that this enzyme controls the temperature-dependent activity of the holin/endolysin cassette (23).”

L107-118 I would argue if you have done through a serial dose series that seems to be enough data here to determine the Ld50 for a set time period eg but not necessarily 3 days post challenge

**Answer**: We carefully considered this point again and used the data of our preliminary infection experiments, which were performed to identify an appropriate infection dose, for a respective calculation. However, we failed due to a too low sample size. To nonetheless address this point, we now mention the LD50 of subcutaneous W22703 infection as published in a previous paper (line 136-139).

L130 perhaps say challenging larvae with LB medium ?

**Answer:** This was accordingly rewritten to “…all larvae survived, corresponding to a challenge of the larvae with LB medium…” (lines 143-144).

L133 still unsure about pARA leakiness can this be explained better can you place a example from another system I think a paper of Guzman ([https://pubmed.ncbi.nlm.nih.gov/7608087/](https://scanmail.trustwave.com/?c=4477&d=qsDE4_IvgAOuOwceLK0FIHZZWxKuXbvI-sPoW-2B9A&u=https%3a%2f%2fpubmed%2encbi%2enlm%2enih%2egov%2f7608087%2f)) highlight basal leakyness and could be referenced

**Answer:** We observed such a leakiness in previous studies (Springer et al., now cited in the manuscript). We would also like to stress that tight regulation to prevent protein expression does not exclude a slight transcription of a gene whose product must not be detectable by a Western Blot. Gene *tccC* exhibited a very low transcriptional level (see Figure S1), confirming that transcription from pBAD is sufficient for complementation.

L168 complemented plasmid carried the deleted genes is this correct? I might have though the complement plasmids carried the intact genes as per line 132-133 – please clarify (I might have miss read)

**Answer:** The complementing plasmids carried the genes as per line 132-133 in the first revision, that is correct. For clarity, this now reads “…all of which carrying recombinant plasmids that complemented the chromosomally deleted genes…(lines 185-186)”

L220 contradicts the statements on Ll167=170 where full complementation is noted ?

**Answer:** We agree, and we indeed obtained a satisfying complementation using this construct in other experiments. Upon re-evaluating the photos, we indeed found antibody signal pointing to TcaA::RFP also in the hemolymph of larvae infected with W22703 ΔHE tcaA::rfp/pACYC-HE. Staining sites are now exemplarily indicated by arrows, and the results were accordingly rewritten to (lines 231-239): “Following tissue section and immunostaining with anti-*Yersinia* antibody and anti-RFP antibody, we detected TcaA in *Yersinia*-rich hemolymph areas of *G. mellonella* 24 h p.i. with W22703 *tcaA*::*rfp* and W22703 ΔHE *tcaA*::*rfp*/pACYC-HE (**Fig. 6B**). In the absence of the lysis cassette, however, TcaA::Rfp was not detected despite the presence of W22703 ΔHE *tcaA*::*rfp* cells*.* To test whether or not the promoter of the lysis cassette is active *in vivo*, we infected *G. mellonella* larvae with strain W22703 P_HE_::*rfp* that harbours a chromosomal transcriptional fusion of *rfp* with the HE promoter. Although this strain densely proliferated within the hemolymph, we failed to stain RFP possibly due to no or weak P_HE_ activity, an inference that is in line with the results of the transcriptome analysis (**Fig. S1B**).”

L248 might be good to link 12-24 hour in potential role of tcca adhesion also what about holY?

**Answer:** We agree and now link that finding with colonization in the discussion, lines 315-318 (see above). Gene holY is only slightly transcribed in vivo, supporting again that cell lysis is not a prerequisite for Tc release. This is now highlighted in lines 389-391, also in response to reviewer 2.

L272 please expand on what the lux operon is so the reader has greater understanding

**Answer:** Upon re-evaluation, we now highlight the upregulation of genes involved in signalling and therefore skipped the phrase on the lux regulon. The sentence now reads: “Intriguely, several genes encoding factors involved in signaling were transcriptionally activated in the invertebrate, including the *lsr* operon responsible for autoinducer 2 import, regulation, and degradation, and *traI* encoding an acyl-homoserine-lactone synthase.“ (lines 289-292).

L275 can we refer to a table etc on the 13 phage genes

**Answer:** We refer now to Table S1 (transcriptome analysis), and the 13 genes are listed in the category phage in the data sheet of downregulated genes.

The discussion a bit hard to decipher in places, specifically how the examples given link with the findings of the study. Please edit a final time to improve clarity.

**Answer:** We substantially modified and shortened the discussion. The examples of toxins released via phage lysis cassette intend to introduce into the potential mechanisms and to adequately cite the groups that first introduced the different release models. This part was shortened and re-ordered to better link to the following discussion on the mechanism underlying the release of the Tc by Yersinia enterocolitica. Please see all changes made in the manuscript in the marked-up file.

L289 majority excreted I might suggest in the absence of TcA the bacteria is unable to be maintain itself within the gut (this might be a better way to word this).

**Answer:** Thanks for this suggestion that was considered accordingly (line 312).

Is l 291 is this correct Pluminescens in the gut – in this respect the nematode typically infects the haemocoel as the first point of entry - please check

**Answer:** This was a misinterpretation of the paper on Photorhabdus, stating that there is a delay ahead of exponential growth. We removed “…as observed also with P. luminescens (39)” from the text. The sentence now reads “This step occurs approximately 12-18 h after ingestion, a delay reflecting the time of the invasion process.” We also removed the sentence “This phenotype is in contrast to an infection of *Manduca sexta* larvae with the entomopathogen *Photorhabdus luminescens*, during which a large fraction of *P. luminescens* cells is found to be associated with the gut.”

L 297 caco 2 gut cells could mention they are human derived

**Answer:** This was clarified to “…human colonic epithelial (Caco-2) cells…”.

L304 what does readouts? mean please clarify

**Answer:** This sentence was modified to “…of yet unprecedented resolution with respect to the molecular details.” (line 300)

L343 possibly list the genes that specifically target insects

**Answer:** We now cite Table S1 where these genes are found in both data sheets in the category virulence factors. For clarity, this now reads: “In addition, the *in vivo* transcriptional pattern revealed the up-regulation of virulence factors mainly involved in hemolysis and iron scavenging, which are probably specifically directed against insects (Table S1).” (lines 351-353)

Table 1 though highlighted in table supp figure 1 I still might suggest highlight relevant TC and lysis genes

**Answer:** Unfortunately, this is not possible as the transcriptional activity of some tc and lysis genes does not match the stringent threshold imposed on the FC values in Table 1. Figure S1B is now cited twice to bring it to the readers`s attention (lines 239 and 264).

Figures are in general well presented
Fig 7 are the scales accurate of are cells observed in Tca minus? Which appear more larger?

**Answer:** We carefully controlled the scale that, however, is correct in all photos. We assume this is owed to cell size variability as now mentioned in the legend of Fig. 7.
